# Promising Experimental Treatment in Animal Models and Human Studies of Interstitial Cystitis/Bladder Pain Syndrome

**DOI:** 10.3390/ijms25158015

**Published:** 2024-07-23

**Authors:** Ju-Chuan Hu, Hong-Tai Tzeng, Wei-Chia Lee, Jian-Ri Li, Yao-Chi Chuang

**Affiliations:** 1 Department of Urology, Taichung Veterans General Hospital, Taichung 407, Taiwan; shelleycain525@gmail.com (J.-C.H.); fisherfishli@yahoo.com.tw (J.-R.L.); 2 Institute for Translational Research in Biomedicine, Kaohsiung Chang Gung Memorial Hospital, Kaohsiung 833, Taiwan; htay11@cgmh.org.tw; 3 Division of Urology, Kaohsiung Chang Gung Memorial Hospital and Chang Gung University College of Medicine, Kaohsiung 833, Taiwan; chuang82@ms26.hinet.net; 4 Department of Post-Baccalaureate Medicine, College of Medicine, National Chung Hsing University, Taichung 402, Taiwan; 5 College of Nursing, Hungkuang University, Taichung 433, Taiwan

**Keywords:** bladder pain syndrome, interstitial cystitis, monoclonal antibody, nanotechnology, regenerative medicine

## Abstract

Interstitial cystitis/bladder pain Syndrome (IC/BPS) remains a mysterious and intricate urological disorder, presenting significant challenges to healthcare providers. Traditional guidelines for IC/BPS follow a hierarchical model based on symptom severity, advocating for conservative interventions as the initial step, followed by oral pharmacotherapy, intravesical treatments, and, in refractory cases, invasive surgical procedures. This approach embraces a multi-tiered strategy. However, the evolving understanding that IC/BPS represents a paroxysmal chronic pain syndrome, often involving extravesical manifestations and different subtypes, calls for a departure from this uniform approach. This review provides insights into recent advancements in experimental strategies in animal models and human studies. The identified therapeutic approaches fall into four categories: (i) anti-inflammation and anti-angiogenesis using monoclonal antibodies or immune modulation, (ii) regenerative medicine, including stem cell therapy, platelet-rich plasma, and low-intensity extracorporeal shock wave therapy, (iii) drug delivery systems leveraging nanotechnology, and (iv) drug delivery systems assisted by energy devices. Future investigations will require a broader range of animal models, studies on human bladder tissues, and well-designed clinical trials to establish the efficacy and safety of these therapeutic interventions.

## 1. Introduction

Interstitial cystitis/bladder pain syndrome (IC/BPS) remains an enigmatic and complex urological disorder, posing significant challenges to clinicians [1]. Previous guidelines for IC/BPS followed a hierarchical model based on the severity of symptoms, advocating for the initiation of conservative interventions, succeeded by oral pharmacotherapy, intravesical administrations, and, in refractory cases, invasive surgical procedures, thereby adopting a multi-lines approach [2]. However, the emerging understanding that IC/BPS represents a paroxysmal chronic pain syndrome, often entailing extra-vesical manifestations, necessitates a departure from such a one-size-fits-all approach [2,3]. Presently, it is acknowledged that IC/BPS is not amenable to curative interventions and lacks a universally effective long-term treatment strategy [1,2,3]. Consequently, current therapeutic paradigms are shifting toward phenotype-based approaches, leveraging stratification systems like UPOINT (Urinary, Psychosocial, Organ-specific, Infection, Neurologic/systemic, Tenderness) and INPUT (Infection, Neurologic/systemic, Psychosocial, Ulcers, Tenderness of muscle), aimed at tailoring treatment regimens to the unique clinical phenotype exhibited by each patient [4,5,6]. IC/BPS can be classified into two distinct forms: Hunner type interstitial cystitis (HIC) and non-Hunner type interstitial cystitis (NHIC). Hunner ulcers are identified through cystoscopy and present as patches of red, inflamed mucosa on the bladder wall, often with small blood vessels radiating to a central scar. These lesions can cause significant bladder pain and reduced bladder capacity. In contrast, patients without Hunner lesions may exhibit glomerulations (small bleeding points) on the bladder wall after hydrodistension, which is referred to as NHIC or BPS [6]. Currently, scientists indicate significant histopathological differences between HIC and NHIC.

The prevalence of interstitial cystitis (IC) varies significantly, ranging from 0.01% to 2.3%, with women being affected approximately five times more often than men, though diagnostic criteria vary [6]. While the exact pathophysiology of IC/BPS remains unclear, the current literature underscores the role of impaired urothelial barrier function. The bladder wall comprises several layers: the mucosa (including the urothelium and lamina propria), muscularis propria (detrusor muscle responsible for bladder contraction and relaxation during urination), and adventitia, which provide structural support. The inner mucosal layer contains the urothelium, with multinucleated umbrella cells at the surface. These cells form a critical barrier that prevents urine from leaking into the underlying tissues. They achieve this by having a specialized apical membrane enriched with uroplakins and tight junctions, maintaining the impermeability and integrity of the blood-urine barrier [7]. The underlying causes of this urothelial impairment remain elusive but may encompass factors such as alterations of urinary microbiome [8] or Epstein–Barr virus infection [9]. This compromised barrier is hypothesized to permit the permeation of toxic urinary constituents into the suburothelial space. Once these toxic agents gain access, they may activate bladder afferent pathways and precipitate inflammatory cascades, manifesting as cytokine overproduction [10]. The resultant inflammatory milieu is believed to be instrumental in the genesis of hallmark symptoms, including bladder-associated pain and urinary urgency. In addition, there are other potential mechanisms by which chronic pelvic pain is induced in IC/BPS. Bladder oversensitivity elicited by substances and/or alterations in urothelial afferent function lowers the threshold for sensory nerve activation in response to peripheral stimuli, resulting in pain sensation [11]. Finally, central nervous system hypersensitization may play a role in the development of IC/BPS. Within the spinal cord, synaptic plasticity and repetitive activation of nociceptors can trigger central sensitization, resulting in altered gene expression within nociceptors and the persistence painful state [12].

**Figure 1:**
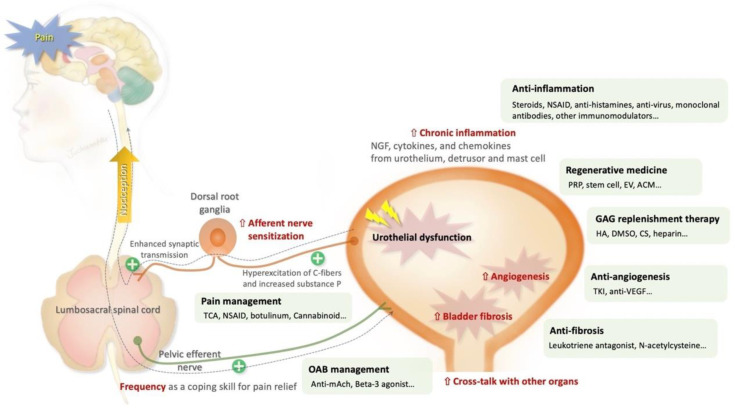
Holistic management based on the complex etiologies and symptomology of interstitial cystitis/bladder pain syndrome (IC/BPS). The pain and urinary symptoms may arise from various pathological processes affecting the afferent and efferent pathways, as well as bladder damage. Based on the symptomology and understanding of pathogenesis, physicians can provide both traditional and novel treatments to alleviate patients’ symptoms.

**Figure 2:**
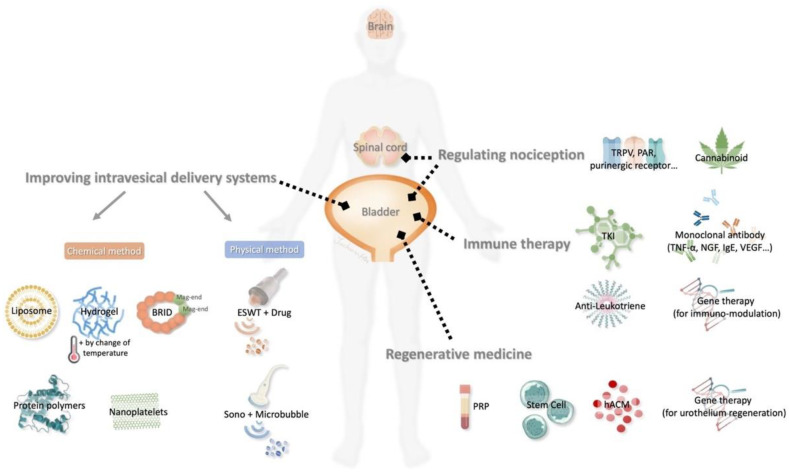
Innovative therapies for IC/PBS. Researchers may explore new modalities to improve drug delivery systems through physical or chemical methods, regulate nociception, use immune modulation, and translate regenerative medicine for the management of IC/PBS.

The holistic management for IC/BPS involves a multifaceted approach that address both intravesical and extravesical pathophysiological pathways as illustrated in Figure 1. Key objectives include reducing local inflammatory responses, modulating the immune system, using regenerative modalities, providing relief from of urinary symptoms and pain, and preventing the development of fibrotic changes associated with chronic cystitis [13]. This comprehensive approach strives to offer a well-rounded treatment strategy that encompasses the intricate etiologies and symptomatology of IC/BPS. Additionally, IC/BPS patients may exhibit overactive bladder (OAB) symptoms [14]. OAB is characterized by a symptom complex including urgency, urinary frequency, and nocturia, with or without urgency urinary incontinence [15]. Although OAB is an idiopathic condition and differs from IC/BPS in pathophysiology, it may present with similar irritable bladder symptoms. The diagnosis of IC/BPS is based on patient-reported symptoms and the exclusion of other diseases with overlapping clinical presentations [14]. Under such circumstances, we review the innovative interventions and advancements for IC/BPS, drawing from evidence from in both animal models and human studies (Figure 2).

## 2. Anti-Inflammation, Anti-Angiogenesis, and Immune Modulation Therapies

Current scientific evidence increasingly implicates the roles of inflammation and angiogenesis in the pathogenesis of IC/BPS [7]. Consequently, several immune-related targets are currently under intensive scrutiny. Potential therapeutic strategies may encompass the suppression of various cytokines, chemokines, growth factors, and mast cells. Additionally, the direct targeting of angiogenic pathways and activation of neurogenic inflammation offer additional theoretically viable approaches for immune-modulating or targeted treatments [7,13].

### 2.1. Monoclonal Antibody Therapy 

It has been established that patients with IC/BPS often display elevated concentrations of inflammatory markers in both bladder tissue and urine. These markers include cytokines like interleukins, as well as nerve growth factor (NGF) and tumor necrosis factor (TNF)-α [16]. Notably, NGF and TNF-α levels are significantly higher in HIC when compared to NHIC [16]. Several clinical trials have aimed to investigate the therapeutic efficacy of monoclonal antibodies targeting these specific markers. Anti-TNF-α antibodies, such as certolizumab pegol [17] and adalimumab [18], have undergone clinical scrutiny. Anti-TNF-α agents have been successfully applied to several autoimmune diseases, including psoriatic arthritis, Crohn’s disease, rheumatic arthritis, juvenile idiopathic arthritis, ankylosing spondylitis, and ulcerative colitis [19]. Similarly, monoclonal antibodies against NGF, including tanezumab [20] and fulranumab [21], have been subjects of investigation. The concise results of clinical trials involved in anti-TNF-α or anti-NGF agents are illustrated in Table 1. Furthermore, trials involving anti-IgE antibodies like omalizumab have also been conducted [22]. However, the outcomes of these trials have been inconsistent. While some studies indicate safety but fail to demonstrate effectiveness, others have been prematurely terminated due to concerns over potential adverse effects. 

#### 2.1.1. Anti-TNF-α Antibodies 

Recent randomized controlled trials examining the use of anti-TNF-α agents, certolizumab pegol and adalimumab, in the treatment of IC/BPS have produced varied outcomes, underscoring the intricate nature of this condition. In a Phase 3 trial with certolizumab pegol, no significant improvement in symptoms was noted at endpoint of week 2 [17]. Nevertheless, significant improvements in the interstitial cystitis symptom index (ICSI), interstitial cystitis problem index (ICPI), pain, and urgency were evident by the 18th week after treatment initiation [17]. On the contrary, a Phase 3 trial involving adalimumab demonstrated significant symptomatic improvement in all participants when compared to their baseline [18]. However, this improvement did not translate into a statistically significant difference when compared to the placebo group at the conclusion of the 12-week treatment period [18]. 

#### 2.1.2. Anti-NGF Antibodies

NGF, a neurotrophic factor, can be produced by bladder detrusor and urothelium, as evidenced by increased levels in the urine and bladder tissue of IC/PBS patients [16]. This points to the sensitization of peripheral and central nerve endings. Therefore, anti-NGF antibodies like tanezumab and fulranumab are gaining attention in clinical research for treating IC/BPS patients [20,21]. Evans et al. reported a short-term reduction in pain and urgency among IC/BPS patients using a single intravenous dose of 200 µg/kg tanezumab at week 6 [23]. However, these effects did not persist in the follow-up periods. In a pooled analysis, Nickel et al. suggested that female IC/BPS patients are more likely to experience pain reduction with tanezumab than with placebo [20]. On the other hand, adverse effects of anti-NGF therapy, such as paresthesia, hyperesthesia, and allodynia, were the subject in clinical trials. Notably, Wang et al. conducted a Phase 2 clinical trial with fulranumab (9 mg) in IC/BPS patients, which was terminated prematurely by the U.S. Food and Drug Administration due to the observed progression of osteoarthritis or osteonecrosis in the participants [21]. Therefore, the researchers proposed that intravesical injection of anti-NGF agents for these IC/BPS patients might help avoid additional systemic adverse effects. 

### 2.2. Anti-Vascular Endothelial Growth Factor (VEGF) Therapy and Hypoxia-Inducible Factor (HIF)-Prolyl Hydroxylase Inhibitors 

Immature vascularization may play a pivotal role in the development of IC/BPS. Consequently, increased concentrations of VEGF and HIF-1α have been detected in the bladder tissue and urine of individuals with IC/BPS, establishing an association between angiogenic processes and the manifestation of urinary frequency and bladder pain [24,25]. In an animal study, researchers reported that VEGF and its receptor (VEGF-R) are urothelial biomarkers of protamine-sulfate-induced denuded bladder associated with hyper-permeability [26]. VEGF may be associated with hyperalgesia experienced by patients and be associated with vascularization with nerve regeneration biologically [24]. Upregulation of HIF-1α and overexpression of VEGF are usually found together in the presence of hypoxia. Hence, scientists may try their efforts on the anti-VEGF and anti-VEGF-R therapies and promote HIF-1α function to treat the IC/BPS by using monoclonal antibodies targeting VEGF, tyrosine kinase inhibitors (TKIs), and HIF-prolyl hydroxylase inhibitors. 

Lai et al. utilized anti-VEGF neutralizing antibodies (10 mg/kg intraperitoneal B20-4.1.1 VEGF mAb) to manage cyclophosphamide (CYP)-induced cystitis in C57BL/6 J mice, in which the systemic blockade of VEGF signaling with anti-VEGF neutralizing antibodies significantly reduced pelvic/bladder nociceptive responses, tested by using von Frey filaments, in CYP-induced cystitis mice [27]. Furthermore, using axitinib (1 mg/kg for 5 days), a selective VEGFR2 TKI, Shin et al. increased the micturition volume and alleviated urothelial denudation, angiogenesis, mast cell infiltration, and fibrosis in hydrochloride-instilled rats [28]. Interestingly, Clayton et al. reported that HIF-prolyl hydroxylase inhibitors (i.e., dimethyloxalylglycine and molidustat) could prevent bladder injury and ameliorate bladder dysfunction in CYP-treated mice [29]. 

Taken together, these animal studies indicate that anti-VEGF-neutralizing antibodies, TKIs, and HIF-prolyl hydroxylase inhibitors could potentially provide valuable approaches for further mitigating bladder and pelvic pain in individuals with IC/PBS. However, to confirm their efficacy, safety, and long-term effects in human IC/BPS patients, these promising findings must be validated through human clinical trials.

## 3. Gene Therapy for Immune Modulation 

Currently, scientists tried to apply gene therapy to IC/BPS treatment. Herpes simplex virus (HSV) vectors is one of the popular carriers involving in targeted gene delivery. In a rat model of resiniferatoxin-induced cystitis, HSV vectors expressing a TNF-α soluble receptor (TNF-α blockade gene) significantly reduced levels of inflammatory cytokines (i.e., IL-1 and IL-6) in the bladder [30]. This approach led to an alleviation of rat bladder overactivity, suggesting a promising avenue for gene therapy in IC/BPS patients. Moreover, the utilization of HSV vectors has extended beyond experimental phases, as they have become the focus of preclinical and clinical trials for managing pain and addressing neurogenic detrusor overactivity [31,32]. 

Another promising gene therapy for urological diseases is the presence of URO-902, a naked plasmid DNA consisting of 6880 base pairs. This DNA encodes the human BK channel α-subunit, a critical modulator of detrusor tone and contraction in bladder smooth muscle cells [33,34]. Phase 1 clinical trials of URO-902 have shown positive results for treating OAB symptoms and improving the quality of life with no reported severe adverse effects, via administrating through intravesical instillation or intra-detrusor injection [33,34]. While specific studies on IC/BPS are currently lacking, the efficacy of URO-902 in OAB treatment suggests that gene therapies with immunomodulatory characteristics could potentially have a substantial role in addressing conditions such as IC/BPS, which were previously considered uncurable. 

## 4. Miscellaneous 

### 4.1. SH2-Containing Inositol-5′-Phosphatase (SHIP) 1 Activator 

SHIP1 is an intracellular protein that serves as a negative regulator of the Phosphoinositide 3-kinase (PI3K) pathway [35]. The PI3K cascade is a central signaling pathway responsible for controlling various cellular processes, including cell proliferation, growth, differentiation, and survival. Precise control of the PI3K signaling pathway is essential to prevent abnormal cell proliferation and the development of cancer [36]. SHIP-1 exerts its negative regulatory role in immune cell activation through interactions with other proteins, particularly Shc, LAT, and members of the Dok protein family [37]. Beyond its enzymatic functions, SHIP1 is also implicated in a range of non-enzymatic, immune-related pathways. Thus, the activation of SHIP1 might yield anti-chemotactic and anti-inflammatory effects. 

An illustrative case of the challenges encountered in the development of immune therapy for IC/BPS is AQX-1125, an oral activator of SHI. In a phase 2 clinical trial [38], a six-week treatment with AQX-1125 led to notable improvements in pain, ICSI, ICPI, and bladder pain IC-symptom score among 37 IC/BPS patients. Despite these promising findings, the subsequent phase 3 trial was unable to replicate the positive outcomes. In this later stage, AQX-1125 failed to demonstrate a more significant therapeutic effect than the placebo across various outcome measures, including pain, frequency, bladder pain/IC symptom score, ICSI, and global response assessment [39].

The disparity observed between the results of the phase 2 and phase 3 trials underscores the intricate nature of IC/BPS and the challenges associated with its study. This underscores the urgent necessity for a deeper comprehension of the differentiation in the inflammatory phenotype and advocates for the consideration of cystoscopy-based classification as a potential approach to enhance the precision of diagnosis and tailor treatment strategies more effectively. Another aspect deserving further investigation is the placebo-nocebo effect, given its potentially substantial impact on treatment outcomes. The AQX-1125 studies could serve as a valuable model for translational medicine, offering insights into how future trial designs could be refined.

### 4.2. Transient Receptor Potential Vanilloid Type 4 (TRPV4) Antagonist 

The TRPV subfamily comprises six members (TRPV1-6). The TRPV1-4 are all activated by heat and nonselective for cations. Elevated bladder TRPV 1, 3, and 4 had been reported in patients and rats with ketamine-induced cystitis [40,41]. Everaerts et al. demonstrated that mice lacking TRPV4 receptors or treated with HC-067047 (an antagonist of TRPV4) can preserve bladder capacity and remain free from urinary frequency, even in the presence of severe CYP-induced cystitis [42]. Furthermore, Charrua et al. observed that the synergic effects of TRPV1 antagonist (RN1734) and TRPV4 antagonist (SB366791) could reverse the bladder hyperactivity of lipopolysaccharide (LPS)-induced cystitis at very low doses. These findings suggest that TRPV4 agonists may hold potential for patients with IC/PBS syndrome [43]. 

### 4.3. Cannabinoids

Cannabinoids have demonstrated urological applications by modulating micturition pathways and spinal cord pain pathways related to urological neuropathic pain control [44]. In the human body, there are two main cannabinoid receptors: CB1 and CB2, part of the G-protein-coupled receptor family [44]. CB1 is a central receptor, while CB2 is peripheral. These receptors, along with endogenous cannabinoids, are found in urothelial cells, urological sensory neurons, bladder detrusor, mucosa, and in the central nervous system related to micturition control [44]. Prior research indicates that CB2 plays a significant role in early inflammatory events, contributing to immune regulation through its expression in various leukocytes, mediating anti-inflammatory effects and immunomodulation [44]. Tambaro et al. demonstrated that administration of JWH015, a selective CB2 agonist, significantly reduced leukocyte infiltration and proinflammatory cytokines in the bladder of CD1 mice [45]. Liu et al. also reported that activating CB2 with its agonist JWH-133 could inhibit mechanical hyperalgesia and alleviate bladder inflammation in CYP-induced cystitis via regulating autophagy in mice [46]. Furthermore, Berger et al. compared the therapeutic effects on LPS-induce cystitis mice among β-caryophyllene (a nature diet sesquiterpenoid and an agonist of CB2), HU308 (the synthetic CB2-selective cannabinoid), and dimethyl sulfoxide (an US Food and Drug Administration approved clinical treatment), in which both nature and synthetic cannabinoids significantly reduced the number of adhering leukocytes in submucosal bladder venules and improved bladder capillary perfusion in intravital microscopy [47]. To explore the specific mechanisms by which cannabidiol relieves inflammation and oxidative stress, Kuret et al. examined how cannabidiol modulates the PPARγ/Nrf2/NFκB signaling pathway in urothelial cells (SV-HUC1) and showed that cannabidiol may decrease TNF-α expression and diminished cellular reactive oxygen species generation. Collectively, cannabinoids may serve as an alternative option for alleviating inflammatory symptoms of IC/BPS [48].

## 5. Regenerative Medicine 

### 5.1. Stem Cells 

Stem cells possess the unique capability to self-renew and differentiate into various lineages, including ectoderm (e.g., epithelium and neurons), mesoderm (e.g., muscle and stroma), and endoderm (e.g., endothelium) [49]. At present, scientists suggest that transplanted stem cells can provide therapeutic benefits via the paracrine release of anti-inflammatory, pro-angiogenic, anti-apoptotic, and anti-oxidative factors [50]. Moreover, these paracrine bioactive factors are thought to enhance the expression of stem cell trafficking genes, leading to the recruitment of endogenous stem cells to damaged tissues [49,50,51]. Currently, the paracrine effects of transplanted stem cells appear more prominent due to their stimulation of host stem cells and adjacent cells [51]. 

Stem cells can be categorized into different cell types, including mesenchymal stem cells (MSCs), embryonic stem cells (ESCs), induced pluripotent stem cells (iPSCs), and hematopoietic stem cells. When compared to ESCs and iPSCs, MSCs exhibit lower tumorigenicity in vivo, making them a safer option for clinical treatments. MSCs, known for their immunomodulatory properties, can be transplanted into immunocompetent recipients without requiring immunosuppressants [52]. When comparing the therapeutic effects of different MSCs in uroplakin II-induced IC rats, which included urine-derived stem cells, adipose-tissue-derived stem cells, bone-marrow-derived stem cells and amniotic-fluid-derived stem cells, Chung et al. [53] found that direct urine-derived stem cell injection into bladder submucosa yielded the most favorable therapeutic outcome. To observe the in vivo behavior of engrafted multipotent MSCs, Yu et al. employed two-proton imaging analysis to visualize the dynamic association between engrafted multipotent MSCs and bladder vasculature in live rats for up to 28 days post-transplantation [54]. This analysis demonstrated the gradual integration of transplanted multipotent MSCs into a perivascular-like structure. Recently, Shin et al. reported that three patients with HIC received cystoscopic submucosal injection of human ESC-derived MSCs (SNU42-MMSCs) [55]. Their study showed encouraging preliminary outcome in pain relief and no recurrence of Hunner ulcers at 12-month post-treatment follow-up. Although the rationale of MSCs in treating IC/BPS is well-established, the immune reaction, low survival rate, and tumorigenicity of stem cells are concerning. To overcome these constraints, researchers have proposed that extracellular vesicle secretion is currently the main mediator of MSC paracrine mechanisms in a cell-free platform [56]. 

### 5.2. Platelet-Rich Plasma (PRP) 

PRP is a blood product with an elevated platelet concentration obtained through centrifugation. Commercial devices simplify its preparation, achieving 2–5 times the baseline platelet concentration, along with clotting factors. PRP contains growth factors, cytokines, and proteins, which affect its therapeutic potential, influenced by leukocytes, activation, fibrin structure, and platelet count [57]. The DEPA (Dose of injected platelets, Efficiency of production, purity of the PRP, Activation of PRP) classification aids in selecting PRP products [57]. PRP contains growth factors and cytokines that can aid in bladder mucosa healing [58]. Platelets play a role in homeostasis through adhesion, activation, and aggregation. Upon activation, platelets release factors promoting coagulation. PRP’s activation releases growth factors and cytokines, including vascular endothelial growth factor, fibroblast growth factor, and interleukin-8 [49]. PRP supports proliferation, migration, differentiation, and angiogenesis in the local environment.

PRP therapy has shown promise in several animal models and pilot clinical studies. In a CYP-induced cystitis rat model, Chen et al. showed that intravesical PRP instillation could improve cystometric parameters and modulate urothelial repair [59]. Chueh et al. reported their findings on the therapeutic effects of PRP for ketamine-induced cystitis, highlighting that PRP therapy for this severe cystitis has anti-inflammatory, anti-fibrotic, antioxidant, angiogenetic, and urothelium regeneration-promoting properties [60]. Based on electron microscopic findings of bladder specimens from IC/BPS patients, Lee et al. demonstrated that repeated intravesical PRP injections effectively improve symptoms in IC/PBS by promoting the recovery of urothelial ultrastructural defects [61]. In a clinical study by Jiang et al., it was suggested that the success rates three months after receiving four consecutive PRP injections was 76% [62]. This study also observed a reduction in urinary biomarkers, including NGF, matrix metalloproteinase-13, and VEGF. After comparing the effects of intravesical PRP and botulinum toxin A (BoNT-A) injections, Jhang et al. concluded that both therapies show similar efficacy in improving IC symptoms [63]. However, patients receiving BoNT-A injection may be at risk of urinary tract infections post- treatment. 

The exact mechanism of PRP in healing IC/BPS remains unclear. PRP might help repair urothelial injuries caused by chronic inflammation and fibrosis, switching inflammation to an anti-inflammatory state and promoting angiogenesis, potentially alleviating neuropathic pain. In summary, intravesical PRP injection holds potential for treating IC/BPS by promoting wound healing, tissue regeneration, and immune modulation, but more research is required to establish guidelines.

### 5.3. Low-Intensity Extracorporeal Shock Wave (Li-ESW) Therapy and Drug Delivery

A shock wave is a continuous sonic wave capable of carrying energy and propagating through a medium. Li-ESWs are believed to possess biological effects that induce anti-inflammatory responses, neovascularization, cell proliferation, and enhance nerve regeneration and cell membrane permeability [64]. Li-ESW has found clinical applications as a non-invasive therapeutic approach for bladder disorders, including OAB [65], underactive bladder [66], and stress urinary incontinence [67]. Then, researchers have embarked on exploring the therapeutic benefits of Li-ESW for patients with IC/PBS. By using a CYP-induced cystitis model, Wang et al. showed that Li-ESW could suppress the bladder pain, inflammation, and overactivity of rats [68]. In a four-week treatment regimen, Chuang et al. [69] reported a reduction in pain among IC/BPS patients using a protocol involving 2000 shocks waves delivered at a frequency of 3 Hz and maximum total energy flow density of 0.25 mj/mm^2^ once a week. Similarly, Shen et al. employed the same treatment protocol in their study and observed a decrease in urinary biomarkers, namely VEGF and IL-9, in IC/BPS patients by the end of week 4 [70]. In a single-arm clinical investigation of IC/BPS, Jhang et al. demonstrated significant improvements in diurnal urinary frequency and ICSI score in IC/BPS patients when applying Li-ESW (3000 pulses, frequency of 3 Hz, and maximum total energy flow density 0.25 mj/mm^2^ per week over an 8-week period) [71]. 

Furthermore, Li-ESW may enhance the delivery of pharmaceutical molecules into cells. Through Li-ESW induction, Chuang and colleagues observed the permeation of Gd-diethylenetriamine pentaacetic contrast medium through the bladder urothelium in rats via magnetic resonance imaging [72]. In a preclinical study focused on OAB [73], the simultaneous use of Li-ESW and botulinum toxin resulted in superior cytometric outcomes, reduced submucosal edema, and diminished inflammatory cell infiltration when compared to the application of Li-ESW alone. This combined approach also notably reduced the levels of malondialdehyde, TNF-α, and IL-6.

However, Jiang and colleagues reported preliminary findings from a clinical study on the effects of Li-ESWT for IC/BPS patients, which did not show statistically significant therapeutic benefits [74]. This lack of consistency may be attributed to the heterogeneity of IC/BPS phenotypes and variations in Li-ESW protocol parameters, such as the timing of Li-ESW and the dosage of BoNT-A instillation. While existing clinical trials have not definitively established the adjunctive value of Li-ESW therapy with BoNT-A, the non-invasive nature and minimal side effect profile of Li-ESW therapy warrant further investigation as part of comprehensive treatment strategies for IC/BPS [64].

## 6. Intravesical Delivery Systems 

The urothelium, a multi-layered epithelium characterized by apical umbrella cells, along with the glycosaminoglycan (GAG) layer, plays a crucial role in separating urine from the underlying bladder wall tissue. In the context of IC/BPS, urothelial barrier dysfunction is implicated in the symptoms of some patients but not universally [75]. This compromised barrier integrity is linked to a range of histopathological changes, altered gene expression profiles, and molecular shifts [1,2]. For the patients who are refractory to conservative modalities or oral treatment, current guidelines broadly endorse a range of intravesical therapeutic strategies as subsequent lines of intervention [2,3]. These encompass GAG replenishment therapies as well as intravesical injection therapies. 

Specifically, GAG replenishment therapy involves the intravesical instillation of agents such as hyaluronic acid, dimethyl sulfoxide, heparin, and lidocaine, aimed at either ameliorating urothelial defects or pain relief, and thereby achieving therapeutic benefits [1]. Intravesical injection therapies encompass the direct administration of agents like botulinum toxin [76] and triamcinolone [77] into the detrusor muscle using specialized endoscopic needles during cystoscopy. Intradetrusor botulinum toxin injection is efficacious in attenuating neurogenic inflammation and facilitating analgesia [78], whereas triamcinolone serves to modulate exaggerated immune responses and mitigate chronic inflammation [77]. 

Both instillation and injection-based treatments are constrained by their transient effectiveness, necessitating repeated therapeutic sessions [79]. Given their relative invasiveness and limited long-term effects, the development of innovative drug delivery systems is of utmost significance. Furthermore, the majority of commonly used pharmaceutical agents, such as hyaluronic acid and heparin, possess hydrophilic properties. This not only hinders their efficient penetration through the urothelium but also makes them susceptible to dilution by urine during their stay within the bladder. Consequently, these pharmacokinetic limitations exacerbate the challenges associated with achieving effective therapeutic outcomes in the management of IC/BPS. Adding to this complexity is the frequent occurrence of OAB symptoms, including urinary urgency and frequency, in IC/BPS patients. The premature expulsion of intravesical medication due to urination before therapeutic effects take hold signifies treatment ineffectiveness. This represents a critical bottleneck that urgently requires innovative approaches in intravesical treatment modalities.

### 6.1. Promising Nanotechnologies in Intravesical Drug Delivery System 

Emerging nano-formulations for targeting medicinal drugs in the field of IC/BPS research encompass a range of innovative nanomaterials, including electromotive drug administration [80,81], three-dimensional and four-printing dimensional intravesical devices [82,83], and lidocaine-releasing intravesical systems [84,85], as well as other novel nanocarrier systems [86,87]. Various nanocarriers, such as amphiphilic copolymers, muco-adhesive formulations, hydrogels, floating systems, and liposomes, have shown differential efficacy in augmenting drug delivery while maintaining sustained therapeutic agent release, particularly in the treatment of bladder diseases like IC/BPS [88], bladder cancer [89], and urinary tract infections [90]. In this context of IC/BPS research, we focused on liposomal formulations, hydrogels, and hyaluronic acid nanoplatelets.

#### 6.1.1. Liposomes

Liposomes are indeed a versatile tool in drug delivery and gene therapy, owing to their unique structure that enables the encapsulation of a diverse range of substances and facilitates their delivery into cells. The concentric phospholipid bilayers create a hydrophobic environment, making them well-suited for transporting lipophilic drugs, while the aqueous core accommodates hydrophilic substances. Furthermore, their ability to enter cells through endocytosis enables more targeted delivery, thereby maximizing therapeutic efficacy while minimizing systemic side effects [91,92].

Researchers have reported that administering intravesical liposomes *per se* once a week for four weeks could enhance symptom scores in patients with IC/PBS [93]. Nevertheless, ongoing clinical trials have not conclusively demonstrated the superiority of liposomal onabotulinumtoxin A over a placebo. It is essential to consider the potential impact of placebo effects in interpreting these results. For example, Chuang and Kuo demonstrated that patients experiencing moderate to severe IC/BPS exhibited significant relief in pain symptoms and notable improvements in questionnaire-based metrics (i.e., ICIS and ICPI) after a single bladder instillation of liposomal onabotulinumtoxin A [94]. However, the therapeutic effect was comparable to that observed in the placebo group. Given the capacity of onabotulinumtoxin A to downregulate the expression of neural growth factors, P2X3 receptors, and vanilloid receptors on C-fibers, it would be advantageous to conduct further clinical trials specifically tailored to IC/BPS patients who manifest a dominant profile of neurogenic inflammation and bladder hypersensitivity [95]. 

While chronic cystitis and IC/BPS have distinct pathophysiologies, studies on chronic cystitis provide valuable insights applicable to the context of IC/BPS [96]. In a rat model of ketamine-induced cystitis, repeated instillation of liposomal onabotulinumtoxin A has demonstrated multifaceted therapeutic benefits. This treatment not only alleviates suburothelial hemorrhage but also promotes the repair of tight junctions within the urothelial barrier [97]. Simultaneously, the treatment leads to a significant reduction in substance P levels and suppresses inflammatory mediators, including IL-6 and TNF-α. Furthermore, liposomal onabotulinumtoxin A instillation inhibits the increased expression of mucosal TRPV1 and detrusor muscarinic acetylcholine receptor 2 [97]. Therefore, using liposomal onabotulinumtoxin A in patients with IC/BPS who primarily exhibit symptoms of nociception and bladder hyperactivity may potentially result in superior treatment efficacy.

Beyond onabotulinumtoxin A, ongoing research is exploring liposomal formulations encapsulating a synergistic combination of NGF antisense oligonucleotides [98] and tacrolimus [99]. In pre-clinical studies with rodent models, liposomal tacrolimus has shown potential efficacy in mitigating symptoms of CYP-induced cystitis [99] and holds therapeutic promise in managing radiation-induced and hemorrhagic cystitis [100]. Future investigations will require a more comprehensive array of human tissue studies and rigorously designed clinical trials to substantiate the efficacy and safety profiles of these therapeutic interventions.

#### 6.1.2. Biodegradable Ring-Shaped Implantable Device (BRID) 

A groundbreaking advancement in intravesical drug delivery has been accomplished with the implementation of a BRID [101]. Comprising numerous drug-containing microcapsules constructed from biodegradable polycaprolactone, the BRID structure is connected by bioabsorbable Polydioxanone sutures. Neodymium magnets are employed at both ends to secure the device, allowing it to automatically adopt a ring-like conformation upon insertion into the bladder. This design maximizes mechanical stability and reduces the likelihood of premature ejection during micturition. In a pre-clinical testing using a swine model [101], BRID demonstrated a sustained residence time in the bladder lasting up to four weeks. It maintained stable urinary concentrations of the incorporated drugs—lidocaine and resiquimod—throughout the entire drug elution process. Notably, the device is entirely bioresorbable, eliminating the need for subsequent invasive removal procedures. While this study was not specifically designed to assess therapeutic implications for IC/BPS and was limited to the mentioned drugs, the potential of BRID as a long-term intravesical delivery system is compelling. Future studies could investigate its effectiveness in the sustained release of agents such as botulinum toxin or tacrolimus for the management of IC/BPS.

#### 6.1.3. Thermosensitive Hydrogels/Protein Polymers

Thermosensitive hydrogels were developed to enhance the properties of nanoplatelets for optimal mucosal adhesion and the gradual release of low-molecular hydrophobic drugs [102,103]. An exemplary illustration of this is TC-3 gel, an innovative reverse-thermal gelation hydrogel that can be combined with various therapeutic agents. Specifically, when TC-3 gel is formulated with onabotulinumtoxin A [104], the mixture maintains a fluid state at room temperature, facilitating easy intravesical instillation. Upon exposure to the higher temperature within the urinary bladder, the gel undergoes a phase transition to a solid state, gradually dissolving in urine. This feature enables a prolonged release profile of onabotulinumtoxin A over an extended period. Clinical assessments involving individuals with OAB have reported significant improvements in the severity of urgency and the frequency of urge incontinence [102]. In patients having IC/BPS, intravesical administration of TC-3 gel- onabotulinumtoxin A mixture yielded significant reductions in pain score, ICSI, and ICPI scores as observed at week 12 post-treatment [105]. Another accomplishment in nanotechnology is the development of semi-synthetic GAG ethers engineered to slow down urinary clearance and enhance therapeutic GAGs with inherent anti-inflammatory and analgesic properties for IC/BPS bladder treatment [106].

#### 6.1.4. Nanoplatelets

For replenishment of the GAG layer on the damaged bladder mucosa of IC/PBS patients, hyaluronans were commonly instilled into the bladder [1]. However, most GAG solutions are rapidly eliminated from the bladder by spontaneous voiding. Therefore, researchers designed a nanomaterial GAG by mixing a polysaccharide grafted with fatty acids and α-cyclodextrin in water [107]. These kinds of hyaluronan nanoplatelet with flattened, hexagonal morphology and hydrophobicity have shown distinctive pharmacokinetic advantages in accelerating diffusion and elevated mucosal adhesion within the urinary bladder, as evidenced by rodent models [105,106]. Preclinical studies further substantiate their anti-inflammatory effect on the bladder epithelium and their capacity for urothelial surface regeneration [108]. Given their promising in vivo results, hyaluronan nanoplatelets have the potential to significantly augment the cost-efficacy of intravesical therapies upon successful translation to clinical trials.

### 6.2. Ultrasound-Mediated Microbubble (USMB) Delivery

Drawing from experiences in exploring intravesical delivery systems of cytotoxic agents for bladder cancer, scientists have suggested utilizing USMB when considering new therapeutic modalities for IC/BPS [109].

The utilization of microbubbles in drug delivery can be categorized into two primary strategies: co-administration of microbubbles alongside the therapeutic agent, where the microbubbles and drugs are distinct entities in the formulation, and drug-encapsulated microbubbles, which involve entrapping the drug within the microbubble itself [109]. These methodologies can be administered either via intravascular systemic injections or localized intra-organ injections. Subsequent ultrasound activation of these microbubbles within the target organ can facilitate oscillation or rupture of the microbubbles, thereby releasing the entrapped drug for therapeutic application [110]. The benefits of implementing USMB delivery in human clinical trials serve dual purposes: firstly, to alleviate the adverse effects linked with oral pharmacotherapy, and secondly, to investigate its potential as an innovative drug delivery mechanism aimed at addressing urothelial dysfunction for IC/BPS.

### 6.3. Bridging the Bench to Bedside Gap

Issues regarding the precise phenotyping of IC/BPS and patient selection persist due to the elusive nature of its pathophysiology, resulting in an absence of a definitive standard of care. Consequently, therapeutic interventions and pharmacological agents, despite success in preclinical animal studies or in vitro human urothelial cell culture assays, often fail in clinical trials, possibly due to inherent patient heterogeneity. Stratifying trial participants into HIC and NHIC cohorts for more nuanced analysis is strongly advocated to bridge the translational gap and improve the applicability of research findings to human therapeutic regimens.

Translating animal and in vitro studies to human trials faces challenges, primarily due to the limited clinical relevance of prevailing animal models. Models inducing acute cystitis, like CYP-induced cystitis, closely resemble hemorrhagic cystitis or HIC rather than the broader spectrum of IC/BPS populations. To enhance translational success, meticulous patient selection criteria must be applied. Additionally, capturing the psychosomatic aspects of IC/BPS, especially prevalent in NHIC, poses difficulties in preclinical investigations [111].

IC/BPS is associated with subjective pain experiences and psychosomatic symptoms, necessitating consideration of the placebo and nocebo effects in clinical trials [112]. These phenomena, influenced by patient expectations, challenge conventional trial design and interpretation. Incorporating active control groups and objective assessment measures such as urinary biomarkers and histological findings could strengthen future trial frameworks for evaluating intervention effectiveness.

## 7. Conclusions

Precision treatment for IC/BPS is hindered by the intricate and multifaceted nature of its underlying pathophysiology. Research in this field operates in a bidirectional continuum. Therapeutic modalities showing promise in preclinical animal models or human bladder tissue assays must undergo rigorous, multi-phase clinical trials to confirm their safety and efficacy (from bench to bedside). Continuous efforts in refining phenotyping methodologies are crucial in treating IC/BPS effectively. Additionally, adopting a multimodal therapeutic approach and regular evaluation are essential for comprehensive patient care in managing IC/BPS.

## Figures and Tables

**Table 1:** Results of monoclonal antibodies in treating patients with interstitial cystitis/bladder pain syndrome.

Antibody	Author (Year)	Medicine (Active/Placebo)	Patient Number	Study Design	Duration of Follow-Up	Route and Dosage	Clinical Outcome	Adverse Events (%)
Anti-NGF	Evans [23] (2011)	Tanezumab	(34/30)	Clinical	16 wks	200 μg/kg IV in single dose	Significantly improved in daily pain score and GRA	Paresthesia (17.6) Hyperesthesia (8.8)
	Nickel [20] (2016)	Tanezumab	(104/104)	Meta- analysis	At week of interest	1. 200 μg/kg IV 2. 20 mg IV 3. 30 mg SC	Significant improvement of pain intensity in patients presenting somatic syndrome	Headache (16.3) Paresthesia (15.4)
	Wang [21] (2017)	Fulranumab	(14/17)	Clinical trial	12 wks	9 mg SC	Efficacy was not demonstrated and this study was terminated prematurely	Rapidly progressing osteoarthritis or osteonecrosis.
Anti-TNF	Bosch [18] (2014)	Adalimub	(21/22)	Clinical trial	12 wks	80 mg SC loading dose and 40 mg/2 wk 400 mg SC/2 wk for 4 times	Similar to placebo effect Significantly improved in GRA ICSI, and urgency at week 18	No severe adverse effect UTI (25) URI (3.6)
	Bosch [17] (2018)	Certolizumab pegol	(28/14)	Clinical trial	18 wks	400 mg SC/2 wk for 4 times	Significantly improved in GRA, ICSI, and urgency at week 18.	UTI (25) URI (3.6)

Abbreviations: GRA: global response assessment, ICSI: interstitial cystitis symptom index, IV: intravenous, NGF: nerve growth factor, SC: subcutaneous, TNF: tumor necrosis factor, URI: upper respiratory infection, UTI: urinary tract infection.
